# Assessing the Health Education Needs of Heart Failure Patients in Saudi Arabia

**DOI:** 10.7759/cureus.67610

**Published:** 2024-08-23

**Authors:** Tahani D Aldawsari, Sarah D Aldawsari, Huda S Alshehri, Zainab M Olwani, Amal H Sayyadi, Kholod A Albeshi, Amnah A Jubran, Abeer S Alenzi, Mona T Alanzi, Wafi S Alenzi, Haya M Alshammari

**Affiliations:** 1 Nursing, King Saud University, Riyadh, SAU; 2 Radiology, Alghad International Colleges, Riyadh, SAU; 3 Nursing, Inaya Colleges, Riyadh, SAU; 4 Nursing, Health Sciences College, Riyadh, SAU; 5 Nursing, Majmaah University, Riyadh, SAU; 6 Nursing, Al-Sabai Institute for Health Training, Riyadh, SAU; 7 Nursing, Hail Health Institute, Hail, SAU

**Keywords:** king fahad medical city, educational challenges, preferred education methods, patient needs, health education, heart failure

## Abstract

Objectives

This study aimed to assess the health education needs of heart failure patients at King Fahad Medical City in Riyadh. It also sought to identify the preferred methods of health education and the challenges these patients face during educational sessions.

Methods

A descriptive, cross-sectional survey study was conducted between January 2024 and June 2024. Data were collected using a self-administered questionnaire, designed based on previous studies and established frameworks. Statistical analysis was performed using SPSS version 21.

Results

The study found that health education is crucial and highly sought after by many Saudi patients. Different patients have varying requirements for health education, with one-on-one sessions led by physicians identified as the most preferred method. However, patients frequently encountered challenges, including unclear medical terminology, insufficient time for questions, and an overwhelming amount of information. Concerns were also raised about the educators’ listening skills and the effectiveness of their educational approach.

Conclusion

To address these challenges, it is recommended that health education needs be integrated into clinic visits, involving all relevant healthcare professionals such as nurses, pharmacists, and physicians. This integration can ensure that patients receive comprehensive knowledge about their conditions, thereby improving their health behaviors and outcomes.

## Introduction

Heart failure (HF) is a clinical syndrome that is the leading cause of hospitalization and death and presents significant challenges for patients and their families. More than 64 million people around the world are affected by HF, and this number continues to rise [1]. Saudi Arabia saw a 20.97% prevalence in HF on the scale set from 1990 to 2019, and according to Joinpoint’s regression analysis, Saudi Arabia saw an average annual percentage change (AAPC) of 0.7% during this period, indicating one of the largest increases [2]. The prevalence of HF is projected to increase by 46% by 2030, and associated healthcare costs are expected to grow exponentially over the next decade [3]. Efforts to reduce the societal, health, and economic burden of HF have thus become a public health imperative [4]. Patients with HF often experience multiple symptoms simultaneously, some synergistic and reinforcing each other to form symptomatic clusters [5]. These groups are influenced by intrinsic and environmental factors [6]. However, the majority of patients with HF struggle to perform the basic tasks required to care for themselves with HF [7]. HF restricts physical and social activities, with approximately 11% to 75% of patients with HF having difficulties with daily activities [8]. In addition, advances in HF treatment and equipment, while improving patient prognosis, also complicate treatment plans, posing significant challenges for patients [9]. Therefore, relying solely on patients to care for themselves is not enough. Caregivers play an important pivotal role in supporting and promoting self-care behaviors in patients with HF; however, adherence to care is typically low among HF patients worldwide [10].

Effective health education interventions for HF patients can theoretically improve their health outcomes, including readmissions, short-term mortality, and quality of life [11]. However, the result of a meta-analysis of 5,264 HF patients showed that self-management interventions were not effective in reducing the rate of readmission [12]. Some studies have indicated that the reduced effectiveness of self-management interventions was caused by the knowledge gap between healthcare professionals (HCPs) and patients [13]. A study reported that less than 10% of all patients who received education at discharge understood what they had learned [14]. In addition, HF patients adhered to their prescribed medication regimens but did not follow recommended behavioral changes, including physical activity and weight control [15]. These results suggest that effective discharge education is necessary to improve self-management among HF patients.

Asiri et al. [16] generally point out that in a one-to-one study, educational clinics were the most preferred method by study participants, preferably delivered by a doctor, he concluded that the importance of the health education service was evident in the participants’ responses, but it was not yet sufficient to attract the majority of attendees to the various clinics at Prince Sultan Military Medical City (PSMMC). Al-Khashan et al. [17] point out that health reforms aiming to increase patients’ participation in decision-making require them to be health-conscious; hence, the importance of health education. This importance stems from understanding potential gender differences in health education needs and preferences. Health education has a significant impact on educational interventions for patients living with coronary artery disease (CAD), as Eckman et al. [18] indicated. They randomly selected 187 patients and key outcome measures included assessment of CAD knowledge, clinical outcomes (weight and blood pressure), and healthy behaviors (diet, exercise, smoking). Functional health literacy was assessed as a potential predictor variable. It was concluded that there was an improvement in the scores of knowledge and health behaviors after both interventions. The importance of health education extends to the development of a strategic plan to train health education professionals while providing effective services, which was classified as very satisfactory in this community [16].

Globally, there is growing interest in evaluating methods for monitoring health services and the quality of healthcare delivery in health institutions [19]. Assessing the quality of health education is a significant obstacle to better interventions and broader recognition of its importance in improving public health [20].

Based on this, health education interventions will be more effective if appropriately targeted to different segments of society according to their preferences and needs. Therefore, it is necessary to engage patients regularly, and frequent assessments must be conducted to prioritize their needs and improve health patterns and required health education. The main objective of this study is to assess the health education needs of HF patients and how to provide them in Riyadh, as well as to understand patients’ preferences regarding health education services.

## Materials and methods

Study design and setting

A cross-sectional survey study was conducted between January 2024 and June 2024 at King Fahad Medical City (KFMC) in Riyadh, Saudi Arabia. KFMC is a prominent healthcare facility offering advanced medical care, including specialized treatment for cardiovascular diseases. The study was carried out in both the outpatient and inpatient cardiac clinics within KFMC, which cater to a broad spectrum of patients with heart conditions from Riyadh and neighboring areas.

Study population and setting

The study targeted HF patients, both inpatients and outpatients, aged 18 and older. The clinics are equipped with state-of-the-art facilities and are staffed by a multidisciplinary team of HCPs specializing in cardiology. These clinics provide comprehensive diagnostic and therapeutic services, making them an ideal setting for assessing patients’ health education needs.

Inclusion and exclusion criteria

Participants were included if they were HF patients who provided verbal consent to participate. Patients were excluded if they declined to participate, had mental or speech disorders that could impair their ability to provide informed consent, or were under 18 years old without an adult guardian.

Ethical considerations

Institutional review board (IRB) approval for the study titled ‘Assessing the Health Education Needs of Heart Failure Patients in Saudi Arabia’ was obtained from the IRB under reference number 24-062E. The submission, dated February 1, 2024, was reviewed and approved in accordance with ICH GCP guidelines. Written informed consent was obtained from all participants, who were fully briefed on the study’s objectives, potential benefits, and risks. Participants were assured of their voluntary participation and their right to withdraw from the study at any time without obligation.

To address ethical concerns, a participant information sheet (PIS) was included on the first page of the questionnaire. This sheet outlined the study’s purpose, potential risks, and benefits, and provided instructions for voluntary participation. By completing the questionnaire, whether online or in printed form, participants implicitly consented to participate, as indicated in the PIS.

The survey was designed to ensure confidentiality by not collecting any identifying information that could link participants to their responses. Upon completion, participants were directed to a secure website where their encrypted responses were stored on a password-protected server, accessible only to authorized members of the research team [21].

Sample size calculation

The minimum expected sample size was determined to be 400 participants, which includes an additional buffer to account for any potential dropout or data deficiencies. This calculation was based on the following parameters [22]:

• Required accuracy (margin of error): 5%

• Study power: 95%

• Participants’ age: Over 18 years

• Expected total number of patients: 383

**Figure 1:**
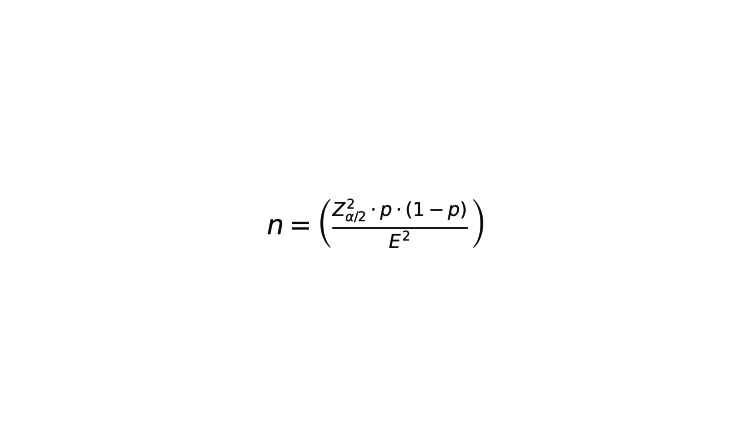
Sample size formula Where: • n: sample size • Z_{\alpha/2}: Z-value associated with the confidence level (for 95%, Z is 1.96) • p: expected proportion (if unknown, 0.5 is commonly used as a conservative estimate) • E: desired margin of error (in this case, 5% or 0.05) Given these parameters, the estimated sample size was calculated to be 400 participants.

To ensure robust study results, the sample size was calculated using the following formula in Figure 1 for proportion measurements:

Sampling technique

The calculated sample size was proportionally distributed across the different areas within KFMC based on the annual rate of attendees in each area. For example, to determine the sample distribution in the outpatient clinics (OPD), we used the following equation: the total number of patients seen in OPD clinics (56,751) divided by the total number of patients in the entire Prince Fahad Medical City (KFMC) (184,474), then multiplied by the sample size (400), as indicated in Table 1.

**Table 1:** Sample size distribution per King Fahad Medical City (KFMC) areas

Strata	N	Sample (%)	Survey forms
Outpatient clinics	56751	31	124
Inpatient clinics	11885	6	24
Primary care	115838	63	252
Total	184474	100	400

Participant enrollment was based on a simple random sampling technique to ensure that all attendees had an equal chance of being included. Participants were selected on a first-come, first-served basis after being informed of the study’s objectives and expected outcomes. They were also assured of full privacy concerning the information obtained. Written consent was obtained from all participants.

Data collection

The design of the questionnaire was based on a review of the relevant literature, including the study by Asiri et al. and the study by Al-Khashan et al. [16,17]. Recognizing that some of the questions included had not been previously tested, a pilot survey was conducted involving 20 participants from KFMC, representing 5% of the sample size. This pilot study aimed to test the questionnaire items and evaluate both content and face validity.

The reliability of the questionnaire was rigorously assessed. Findings from the pilot study informed further refinement, including rephrasing unclear questions and incorporating feedback from HCPs on the health education process. These adjustments ensured the questionnaire’s relevance and clarity.

Subsequently, the questionnaire was translated into Arabic and underwent back-translation by a bilingual individual unfamiliar with the original English version. Any discrepancies in translation were resolved through discussion between the translators and the researchers.

The piloting phase lasted one month. The final version of the questionnaire includes sections on sociodemographic characteristics, the availability and provision of health education services, patient preferences, and challenges for these services.

Procedures regarding the survey application

The survey was administered to participants using an indirect distribution method, specifically through electronic distribution via email. The research survey utilized a five-point scale with the following response options: strongly agree, agree, neutral, disagree, and strongly disagree.

To determine the length of the five-point scale cells (lower and upper bounds) used in the research axes, the range (5 - 1 = 4) was calculated, then divided by the number of scale cells to obtain the correct cell length: (4/4 = 1.00). This value was then added to the lowest value on the scale (or the starting point, which is 1) to determine the upper limit of this cell. Thus, the length of the cells became as indicated in Table 2.

**Table 2:** Five-point scale ranges

Scale Description	Scale Range
Very high	5.0-4.21
High	4.20-3.41
Medium	3.40-2.61
Low	2.60-1.81
Very low	1.80-1.00

Statistical analysis

A total of 400 sample sizes was included in the final analysis. SPSS version 21 was used for data entry and evaluation. The frequency and percentages were obtained using descriptive statistics. Using the K-square test, the relationship between categorical variables was investigated.

## Results

Demographic analysis

The demographic analysis in Table 3 for HF patients at KFMC in Saudi Arabia reveals significant insights into the patient population.

**Table 3:** Descriptive statistics of sociodemographic characteristics of participants (N=400)

Variables	Frequency	Percentage
Age		
24-18 years	4	1
31-25 years	5	1.5
38-32 years	13	2.5
39- 45 years	77	19.3
46 < years	301	75.3
Gender		
Male	303	75.8
Female	95	23.7
Preferable not to declare	2	0.5
Nationality		
Saudi	325	81.3
Others	75	18.8
Marital Status		
Single	72	18
Married	323	80.8
Divorced	5	1.2
Education		
Illiterate	5	1
Intermediate	8	2
Secondary	151	37.8
Diploma	172	43
Bachelor	62	15.5
Graduate	2	0.1
Occupation		
Student	2	0.3
Employed	306	76.5
Not working	15	2
Retired	75	18.8
Other	2	0.3
Place of Residence		
Riyadh	329	82.3
Other	71	17.8

The mean age of participants (N=400) was calculated to be 47.41 years, indicating the average age within the sample. This value, derived from midpoint estimates and a conservative assumption for the “46 years and above” category, provides an overall view of the age distribution. The standard deviation of 5.23 years suggests a relatively narrow dispersion of ages around the mean, implying that most participants’ ages are closely clustered. The median age of 50.0 years further supports this, showing that at least half of the participants are aged 50 or older. Notably, the interquartile range (IQR) was observed to be 0 years, which indicates a very tight clustering of ages within the sample, likely confined to the “46 years and above” category.

The gender distribution reveals that men constitute the majority at 75.8% (303 participants), while women make up 23.7% (95 participants). Most participants are Saudi nationals, accounting for 81.3% (325 individuals), with the remaining 18.8% (75 individuals) from other nationalities. Marital status indicates that a significant portion of participants are married, at 80.8% (323 individuals), while 18% (72 individuals) are single, and 1.2% (5 individuals) are divorced. Education levels vary, with the largest groups having secondary education (37.8%, 151 participants) and diploma certificates (43%, 172 participants), while those holding bachelor’s degrees constitute 15.5% (62 participants), and illiterate individuals make up 1% (5 participants). Employment status shows that 76.5% (306 individuals) are employed, 18.8% (75 individuals) are retired, and 2% (15 individuals) are unemployed. Most participants reside in Riyadh, comprising 82.3% (329 individuals) of the sample, while 17.8% (71 individuals) live in other areas.

Axes questionnaire

Provision of Health Education Services

Table 4 provides an in-depth overview of how health education services are provided to HF patients at KFMC. The table includes data on whether patients have received health education advice, the providers of this education, the methods used to deliver it, and the level of patient satisfaction.

**Table 4:** Descriptive statistics for the provision of health education services

Sentences	Frequency	Percentage (%)	Chi-square (χ²)	P-value	DF
Have You Received Health Education Advice?					
Yes	322	80.5	148.84	0.0000	1
No	78	19.5			
Health Education Providers					
Doctor	302	75.5	834.6	0.0000	5
Health educator	15	2.5	39.8	0.0000	5
Nurse	5	1.0	0.0	0.0000	5
Dietitian	3	0.3	56.8	0.0000	5
Social worker	-	-	-	-	-
Pharmacist	-	-	-	-	-
Other patients	69	17.3	0.094	0.9998	5
Other	5	1.0	56.8	0.0000	5
Health Education Tips Methods					
Health education clinic (one-to-one)	315	78.8	690.3	0.0000	4
Group education	4	0.8	72.2	0.0000	4
Printed educational materials	3	0.5	74.11	0.0000	4
Educational information in the waiting room	69	17.3	1.5125	0.824	4
Other	9	1.2	63.01	0.0000	4
Satisfaction with Health Education					
Fully satisfied	318	79.5	255.66	0.0000	2
Somewhat satisfied	77	19.3	23.15	0.00012	2
Not satisfied	5	1.0	123.56	0.0000	2

Out of the 400 respondents, 322 (80.5%) reported receiving health education advice, while 78 (19.5%) did not. The chi-square test revealed a highly significant association between receiving health education and overall satisfaction (χ² = 148.84, p < 0.0001).

Physicians were the primary source of health education, with 302 patients (75.5%) receiving information from them. Other sources were less common, with health educators (2.5%), nurses (1.0%), and dietitians (0.3%) playing minimal roles. The chi-square analysis for these variables also indicated significant differences (p < 0.0001 for each).

One-to-one education in a clinic setting was the predominant method, used by 315 patients (78.8%). Group education and printed materials were notably less common, at 0.8% and 0.5% respectively. A majority of patients (79.5%) reported being fully satisfied with the health education they received, while 19.3% were somewhat satisfied, and 1.0% were not satisfied at all. The chi-square tests confirmed that satisfaction levels were significantly associated with the provision and method of education (χ² = 255.66, p < 0.0001).

Patients Preference for Health Education Services

Table 5 provides insights into patient preferences for health education services at KFMC. It includes data on the perceived need for health education, preferred providers, and preferred methods of education.

**Table 5:** Descriptive statistics of patients’ preference for health education services

Sentences	Frequency	Percentage (%)	Chi-square (χ²)	P-value	DF
The Need for Such Health Education Services					
Yes	330	82.5	169	0.0000	1
No	70	17.5			
Preferred Health Education Provider					
Doctor	318	79.5	255.66	0.0000	2
Health educators	12	3.0	110.41	0.0000	2
Others	70	17.5	30.08	0.0000	2
Preferred Method of Health Education					
Health education clinic (one-to-one)	247	61.8	218.66	0.0000	3
Group education	75	18.8	6.03	0.11016	3
Printed educational materials	-	-	-	-	-
Health education in the waiting room	-	-	-	-	-
Media (internet or social media)	6	1.5	87.86	0.0000	3
Out of stock	-	-	-	-	-
Other	70	17.5	8.74	0.03296	3

Out of the surveyed population, 330 patients (82.5%) recognized the need for health education services, while 70 patients (17.5%) did not see the need. The chi-square analysis (χ² = 169, p < 0.0001) indicates a highly significant association between the perceived need for education and patient preferences.

A significant majority of patients (79.5%) preferred receiving health education from doctors. Health educators were preferred by a smaller proportion (3.0%), and 17.5% of patients preferred other providers. The chi-square test showed significant differences in preferences (χ² = 255.66, p < 0.0001 for doctors; χ² = 110.41, p < 0.0001 for health educators; χ² = 30.08, p < 0.0001 for others).

The one-to-one health education clinic was the most preferred method, chosen by 247 patients (61.8%). Group education was preferred by 18.8%, and media (internet or social media) was preferred by 1.5%. Additionally, 17.5% of patients preferred other unspecified methods. The chi-square test revealed significant associations between the method of education and patient satisfaction (χ² = 218.66, p < 0.0001 for one-to-one clinics; χ² = 87.86, p < 0.0001 for media; χ² = 8.74, p = 0.03296 for other methods). Notably, the preference for group education was not statistically significant (χ² = 6.03, p = 0.11016), suggesting that while it is valued, it might not be as impactful or widely accepted as individual consultations.

Main Health Education Needs

Table 6 offers a detailed examination of the primary health education needs of HF patients, highlighting their key priorities and concerns.

**Table 6:** Descriptive statistics of main health education needs

Sentences	Mean	St.D	Level
Medical information about your disease.	4.450	0.532	Very high
Living with the disease	4.428	0.495	Very high
Prevention of complications of the disease	4.405	0.540	Very high
Lifestyle modification (e.g. diet, exercise, weight loss)	4.430	0.548	Very high
Taking special medications	4.425	0.524	Very high
Preventive periodic check-ups	4.432	0.530	Very high
How to prevent diseases	4.412	0.531	Very high
Use of medical instruments	4.418	0.538	Very high
Unhealthy practices (e.g. smoking)	4.395	0.616	Very high

The results reveal significant insights into the primary health educational needs of patients at KFMC. Each area of need was rated on a scale from 1 to 5, with higher scores indicating greater importance.

The category medical information about your disease received the highest average score of 4.450 (standard deviation = 0.532). Similarly, those living with the disease scored highly, with an average of 4.428 (standard deviation = 0.495). The topic of prevention of disease complications had an average score of 4.405 (standard deviation = 0.540). Lifestyle modification (e.g., diet, exercise, weight loss) received an average score of 4.430 (standard deviation = 0.548). Medication adherence was evaluated with an average score of 4.425 (standard deviation = 0.524). Preventive check-ups had an average score of 4.432 (standard deviation = 0.530).

How to prevent diseases scored an average of 4.412 (standard deviation = 0.531), while the use of medical devices had an average score of 4.418 (standard deviation = 0.538). Unhealthy practices (e.g., smoking) received the lowest average score of 4.395 (standard deviation = 0.616) among the assessed areas.

Health Education Challenges

The results presented in Table 7 address the critical challenges faced by HF patients during health education sessions. 

**Table 7:** Challenges of health education for heart failure patients

Sentences	Mean	St.D	Level
Unexplained or understandable medical terms	3.290	1.453	Medium
Insufficient time to answer questions.	3.240	1.483	Medium
Different language education	2.450	1.404	Low
Inadequate listening by the education.	3.228	1.475	Medium
Too much information.	3.248	1.482	Medium
Difficulty understanding information.	3.225	1.480	Medium
Inconsistency of information provided by education service providers.	2.826	1.334	Medium
Speed during education	3.227	1.468	Medium
The place is not suitable for education.	3.222	1.467	Medium
Approach or style of education providers.	3.258	1.462	Medium

The challenges are classified on a scale, with mean scores and standard deviations indicating the perceived difficulty level and variability of responses, respectively.

Regarding unexplained or understandable medical terms, the mean score is 3.290, with a standard deviation of 1.453. This indicates that the use of medical terms that are either unexplained or difficult to understand is one of the most significant challenges. Insufficient time to answer questions has a mean score of 3.240, with a standard deviation of 1.483. Different language education represents a lower-level challenge with a mean score of 2.450, though it remains a significant barrier for non-native speakers.

Patients also face a medium-level challenge in inadequate listening by education, with a mean score of 3.228. Too much information is considered a medium-level difficulty with a mean score of 3.248, and difficulty understanding information has a mean score of 3.225. Inconsistency of information provided by education service providers is rated as a medium challenge, with a mean score of 2.826 and a standard deviation of 1.334.

Speed during education is a medium-level challenge with a mean score of 3.227 and a standard deviation of 1.468. This is followed by **the place is not suitable during education, with a mean score of 3.222 and a standard deviation of 1.467, and approach or style of education providers, with a mean score of 3.258 and a standard deviation of 1.462.

## Discussion

The study sample consisted of 400 individuals, predominantly male, over the age of 47, with the vast majority being employed and educated. Most patients had received some form of health education, primarily delivered by doctors in health education clinics.

The results of this study provide a comprehensive overview of health education services for HF patients, revealing significant insights into patient preferences, needs, and challenges. A notable finding is that 80.5% of patients reported having received health education advice, emphasizing the crucial role that educational interventions play in managing chronic conditions like HF. This high rate of reported education aligns with the emphasis placed on patient education in HF management, as highlighted in prior studies [16, 17].

The data shows a clear preference for doctors as the primary providers of health education, with 75.5% of patients favoring them over other professionals. This preference reflects the trust and confidence patients place in their primary healthcare providers and supports findings from previous research that underscore the importance of direct, personalized communication in effective health education [15]. Health education clinics offering one-to-one interactions were also highly preferred, with 78.8% of patients selecting this method. This preference for individualized education is consistent with studies that highlight the benefits of tailored, one-on-one educational interventions for improving patient outcomes [12].

The study also indicates a high level of satisfaction with health education services, with 79.5% of patients expressing full satisfaction. This finding suggests that current educational practices are generally effective in meeting patient needs, although there remains room for enhancement. Previous research has shown that patient satisfaction with educational interventions is closely linked to the quality and clarity of information provided [11].

In terms of health education needs, patients rated several areas as “Very high” in importance, including medical information about their disease, lifestyle modifications, and prevention of complications. These findings are consistent with existing literature that emphasizes the need for comprehensive education covering various aspects of disease management [18]. Addressing these needs effectively can lead to better self-management and improved health outcomes for HF patients.

The study also identified challenges in health education, such as difficulties with understanding medical terms, insufficient time for questions, and the pace of education. These challenges are consistent with previous studies that have highlighted the need for clear, accessible information and adequate time for patient-provider interactions to ensure effective learning [16].

While the study provides valuable insights, it is important to acknowledge its limitations. The findings are specific to the patient population at KFMC and may not fully represent other settings or regions. Additionally, incorporating qualitative insights into patient experiences could provide a more comprehensive understanding of the nuances in patient preferences and challenges.

The study reinforces the importance of personalized, clear, and comprehensive health education for HF patients. The findings are consistent with existing literature and underscore the need for continued improvement in educational strategies to enhance patient self-management and health outcomes. Future research should explore these aspects further, potentially incorporating qualitative data to enrich our understanding of patient needs and preferences in diverse healthcare settings.

## Conclusions

Based on the findings, it is evident that health education needs are significant and widely recognized among Saudi patients. Thus, it can be concluded that patients have varying and diverse requirements for health education, which must be addressed through tailored approaches. It was found that one-to-one health education clinics providing medical instruction by physicians are the most preferred way to communicate health education. Therefore, it is proposed to integrate health education needs during clinic visitation to patients to both nursing and pharmacists and everything that is directly related to the patient. The physician, nurses, pharmacists, or everyone involved in the medical field should give patients complete and adequate knowledge about their diseases and encourage them to change their lifestyle as this can lead to improved health behaviors and therefore improving results.

## Figures and Tables

**Figure 2:**
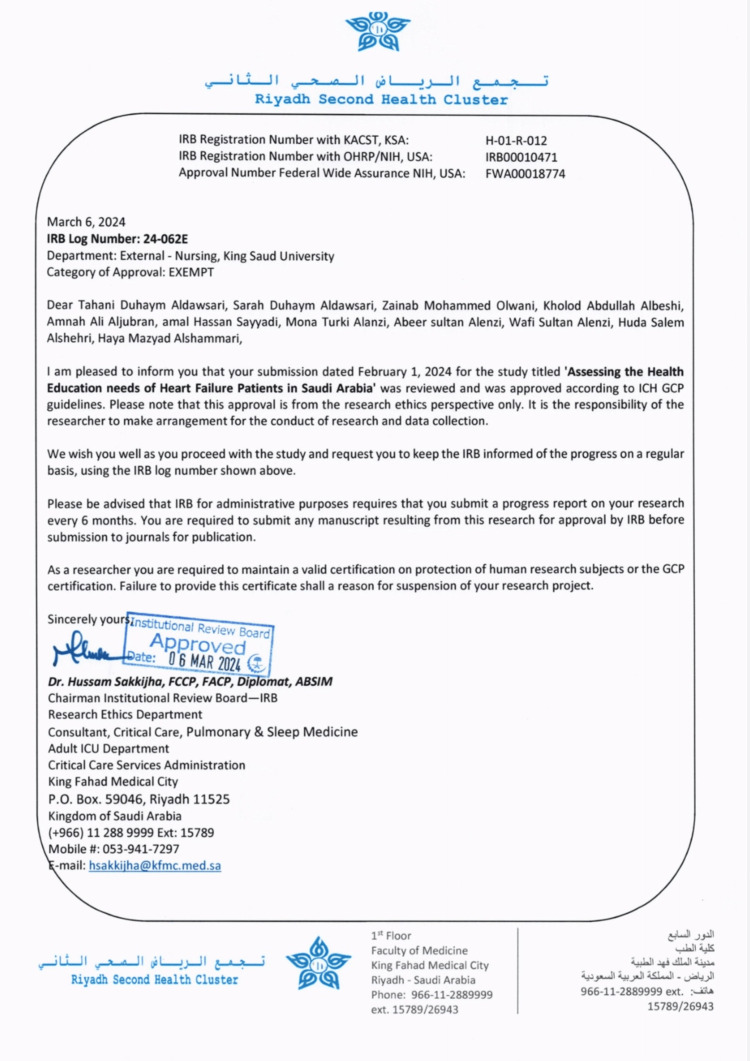
A copy of the informed consent

**Figure 3:**
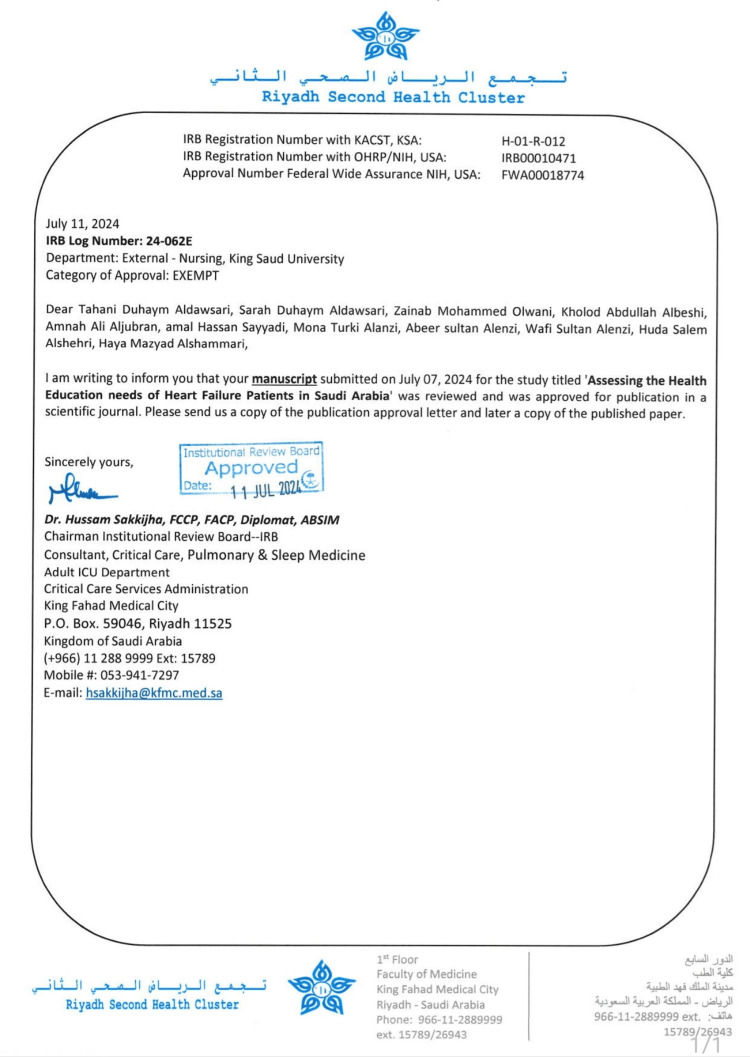
A copy of the approval for publication
